# Clinical Significance of Serum Soluble TNF Receptor I/II Ratio for the Differential Diagnosis of Tumor Necrosis Factor Receptor-Associated Periodic Syndrome From Other Autoinflammatory Diseases

**DOI:** 10.3389/fimmu.2020.576152

**Published:** 2020-10-14

**Authors:** Junko Yasumura, Masaki Shimizu, Tomoko Toma, Masato Yashiro, Akihiro Yachie, Satoshi Okada

**Affiliations:** ^1^Department of Pediatrics, Hiroshima University Graduate School of Biomedical and Health Sciences, Hiroshima, Japan; ^2^Department of Pediatrics, Graduate School of Medical Sciences, Kanazawa University, Kanazawa, Japan; ^3^Department of Pediatrics, Okayama University Hospital, Okayama, Japan

**Keywords:** familial Mediterranean fever, Kawasaki disease (KD), soluble tumor necrosis factor receptor, systemic juvenile idiopathic arthritis (sJIA), tumor necrosis factor receptor associated periodic syndrome (TRAPS)

## Abstract

**Objectives:** Genetic analysis of *TNFRSF1A* can confirm the diagnosis of tumor necrosis factor receptor-associated periodic syndrome (TRAPS), but interpretation of the pathogenesis of variants of unknown significance is sometimes required. The aim of this study was to evaluate the clinical significance of serum soluble tumor necrosis factor receptor type I (sTNFR-I)/II ratio to differentiate TRAPS from other autoinflammatory diseases.

**Methods:** Serum sTNFR-I and sTNFR-II levels were measured using an enzyme-linked immunosorbent assay in patients with TRAPS (*n* = 5), familial Mediterranean fever (FMF) (*n* = 14), systemic juvenile idiopathic arthritis (s-JIA) (*n* = 90), and Kawasaki disease (KD) (*n* = 37) in the active and inactive phase, along with healthy controls (HCs) (*n* = 18).

**Results:** In the active phase, the serum sTNFR-I/II ratio in patients with s-JIA, KD, and FMF was significantly elevated compared with that in HCs, whereas it was not elevated in patients with TRAPS. In the inactive phase, the serum sTNFR-I/II ratio in patients with s-JIA and FMF was significantly higher compared with that in HCs, and the ratio was lower in TRAPS patients than in patients with s-JIA and FMF.

**Conclusions:** Low serum sTNFR-I/II ratio in the active and inactive phase might be useful for the differential diagnosis of TRAPS and other autoinflammatory diseases.

## Introduction

Tumor necrosis factor receptor-associated periodic syndrome (TRAPS) is an autosomal dominantly inherited autoinflammatory disease caused by mutations in TNFRSF1A (1). Symptoms of TRAPS include recurrent fever, abdominal pain, myalgia, exanthema, arthralgia/arthritis, and ocular involvement. Clinical features and laboratory parameters in patients with TRAPS and other autoinflammatory diseases, including systemic juvenile idiopathic arthritis (s-JIA), Kawasaki disease (KD), and familial Mediterranean fever (FMF), tend to overlap. These diseases share clinical manifestations such as fever, rash, and arthritis, as well as laboratory findings such as elevated inflammatory markers. Furthermore, there are no definitive biomarkers for these diseases, making the diagnosis difficult. Genetic analysis of TNFRSF1A can confirm the diagnosis of TRAPS, but interpretation of the pathogenesis of variants of unknown significance is sometimes required. Although the pathogenesis of TRAPS remains unknown, low levels of serum soluble tumor necrosis factor receptor type I (sTNFR-I) in TRAPS patients have been reported (1).

In this study, we aimed to demonstrate that the serum sTNFR-I/II ratio may be useful for differentiating TRAPS from other autoinflammatory diseases including FMF, s-JIA, and KD. We measured serum sTNFR-I and sTNFR-II levels in patients with these autoinflammatory diseases and compared them between each disease.

## Materials and Methods

### Participants

Five TRAPS patients from three families, fourteen FMF patients, 90 s-JIA patients, 37 KD patients, and 18 healthy controls (HCs) were enrolled in this study. Two patients in one family, who we reported previously (2), had a T50M (p.Thr79Met) heterozygous mutation in *TNFRSF1A* and two in another family had a C43R (p.Cys72Arg) heterozygous mutation and one in another family, previously reported (3), had a C30Y (p.Cys59Tyr) heterozygous mutation in the same gene. The initial diagnosis of two of the patients with T50M or C30Y mutation were s-JIA, while that in one of the patients with a C43R mutation was FMF. All FMF patients had a mutation in exon 10 of *MEFV* (thirteen patients with M694I, one with M694V). The diagnosis of s-JIA was based on the International League of Associations for Rheumatology criteria (4). The diagnosis of KD was based on the classic clinical criteria as follows: fever persisting for at least 5 days, changes in extremities (acute phase: erythema of palms and soles, and edema of hands and feet; subacute phase: periungual peeling of fingers and toes in weeks 2 and 3), polymorphous exanthem, bilateral bulbar conjunctival injection without exudate, changes in lips and oral cavity (erythema, cracked lips, strawberry tongue, diffuse injection of oral and pharyngeal mucosae), and cervical lymphadenopathy (≥1.5-cm diameter) (5). The classic diagnosis of KD was based on the presence of ≥5 days of fever and ≥4 of the five principal clinical features (5).

The criteria for the active phase of TRAPS, FMF, and s-JIA are defined as follows: fever, rash, arthritis, and serositis along with increased serum C-reactive protein (CRP) levels. The criteria for the inactive phase on medication include no clinical symptoms that can be seen in the active phase as well as normal CRP levels. Serum samples were collected from three patients with TRAPS, 8 with FMF, 90 with s-JIA, and 33 with KD in the active phase. Serum samples were also collected from five patients with TRAPS, 10 patients with FMF, 33 patients with s-JIA and 6 patients with KD in the inactive phase. The clinical characteristics of these patients in the active phase are shown in Table 1. All patients with s-JIA and KD had fever, but one patient with TRAPS and one patient with FMF had no fever in the active phase. Most patients with s-JIA and KD had rash, and most patients with TRAPS and FMF had serositis. Only one patient with TRAPS was treated with a low dose of prednisone. The patients with FMF, KD, and s-JIA in the active phase received no treatments including prednisone, colchicine, immunosuppressants, and biologics.

**Table 1:** Clinical characteristics and treatment of enrolled patients in the active phase.

	**TRAPS**	**FMF**	**s-JIA**	**KD**
Number of patients (n)	3	8	90	33
Median age (IQR)	8 (3–33)	34 (19–45)	1 (0–3.5)	2 (0–3)
Sex (M, F)	1, 2	2, 6	47, 43	18, 15
**CLINICAL SYMPTOMS**				
Fever	2 (67%)	7 (87.5%)	90 (100%)	33 (100%)
Rash	0 (0%)	0 (0%)	67 (74.4%)	28 (84.8%)
Arthralgia/Arthritis	1 (33%)	0 (0%)	61 (67.8%)	0 (0%)
Conjunctivitis	1 (33%)	0 (0%)	0 (0%)	29 (87.9%)
Serositis	2 (66%)	8 (100%)	8 (8.9%)	0 (0%)
**LABORATORY FINDINGS**				
CRP (mg/dL), median (IQR)	14.1 (6.0–16.1)	3.6 (0.8–13.0)	10.1 (5.7–15.2)	8.7 (4.2–12.2)
**TREATMENTS**				
PSL (n) (mg/kg/day)	1 (0.15)	0	0	0

*IQR, interquartile range; TRAPS, tumor necrosis factor receptor-associated periodic syndrome; FMF, familial Mediterranean fever; s-JIA, systemic juvenile idiopathic arthritis; KD, Kawasaki disease; CRP, C-reactive protein; PSL, prednisolone*.

This study was approved by the Institutional Review Board of Kanazawa University. All participants provided written informed consent. The study was performed in accordance with the ethical standards laid down in an appropriate version of the 1964 Declaration of Helsinki.

### Quantification of Serum Cytokines

Sera were extracted from blood samples, divided into aliquots, frozen, and stored at −80°C until analysis. Serum levels of sTNFR-I and sTNFR-II were measured using a commercial enzyme-linked immunosorbent assay (ELISA) according to the manufacturer's instructions (R&D Systems, Inc., Minneapolis, MN, USA).

### Statistical Analysis

Statistical analysis was performed using GraphPad Prism 7 software (GraphPad, San Diego, CA, USA). Serum sTNFR-I and sTNFR-II levels and sTNFR-I/II ratio were presented as the median and interquartile range (IQR). Comparisons between several groups were performed using one-way analysis of variance with Tukey's multiple comparisons test. A *P*-value of < 0.05 was considered statistically significant.

## Results

### Comparison of Serum sTNFR-I and sTNFR-II Levels and sTNFR-I/II Ratio in TRAPS and Other Autoinflammatory Diseases in the Active Phase

**Figure 1:**
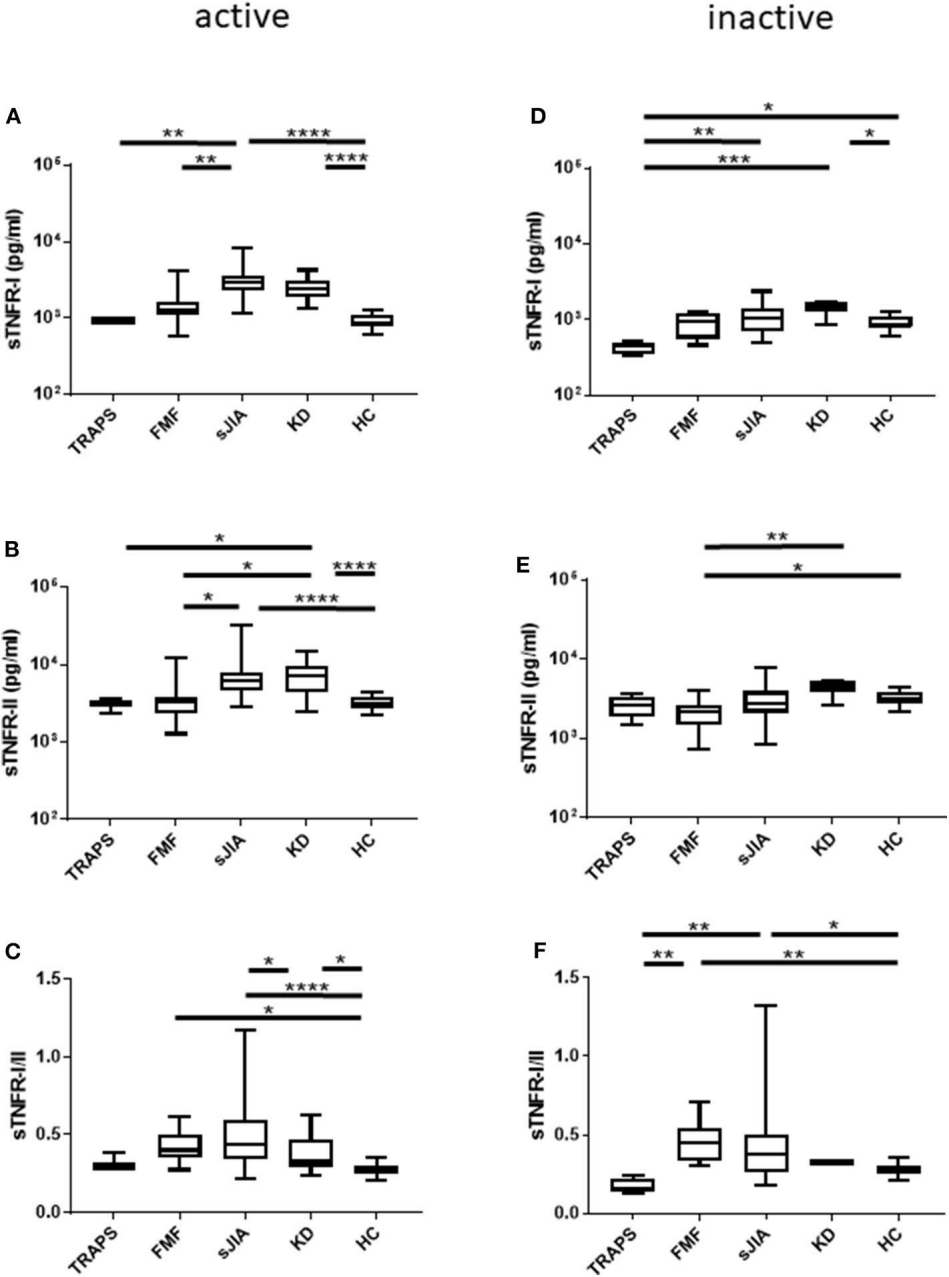
Serum sTNFR-I and sTNFR-II levels and sTNFR-I/II ratio in different patient groups in the active phase and inactive phase. **(A)** sTNFR-I and **(B)** sTNFR-II serum levels and **(C)** sTNFR-I/II ratio in different patient groups in the active phase. Serum levels of **(D)** sTNFR-I and **(E)** sTNFR-II and **(F)** sTNFR-I/II ratio in the inactive phase of different patient groups. Bars represent median values. Statistically significant differences between each patient group are shown as *P < 0.05, **P < 0.01, ***P < 0.001, and ****P < 0.0001. TRAPS, tumor necrosis factor receptor-associated periodic syndrome; FMF, familial Mediterranean fever; s-JIA, systemic juvenile idiopathic arthritis; KD, Kawasaki disease; HC, healthy control.

We measured serum sTNFR-I and sTNFR-II levels in patients with TRAPS in the active phase and compared our findings with those observed in FMF, s-JIA, and KD patients and HCs. As shown in Figure 1A and Table 2, serum sTNFR-I levels were significantly elevated in the active phase in patients with s-JIA (median, 2,900 pg/mL; IQR 2,240–3,563) (*p* < 0.0001) and KD (median, 2,400 pg/mL; IQR 1,860–3,160) (*p* < 0.0001) compared with HCs (median, 835 pg/mL; IQR 795–1,083). Serum sTNFR-I levels were significantly elevated in the active phase in patients with s-JIA compared with FMF (median, 1,260 pg/mL; IQR 1,113–1,635) (*p* < 0.01) and TRAPS (median, 920 pg/mL; IQR 890–1,000) (*p* < 0.01). However, serum sTNFR-I levels in patients with TRAPS were not elevated compared with those in HCs and were significantly lower compared with those in patients with s-JIA. Serum sTNFR-I levels in patients with TRAPS were also lower compared with those in patients with FMF, although this was not statistically significant.

**Table 2:** Serum sTNFR-I and sTNFR-II levels and sTNFR-I/II ratio in each autoinflammatory disease.

	***N***	**sTNFR-I (pg/ml) median (IQR)**	**sTNFR-II (pg/ml) median (IQR)**	**sTNFR-I/II ratio median (IQR)**
**ACTIVE PHASE**				
TRAPS	3	920 (890–1000)	3100 (2330–3550)	0.297 (0.282–0.382)
FMF	8	1260 (1113–1635)	3340 (2375–3858)	0.401 (0.349–0.497)
s-JIA	90	2900 (2240–3563)	6250 (4550–7963)	0.440 (0.342–0.597)
KD	33	2400 (1860–3160)	7250 (4410–9390)	0.327 (0.240–0.469)
**INACTIVE PHASE**				
TRAPS	5	444 (350–495)	2580 (1835–3350)	0.156 (0.136–0.224)
FMF	10	930 (557–1185)	2130 (1475–2638)	0.451 (0.334–0.541)
s-JIA	33	1040 (685–1380)	2750 (2090–3925)	0.379 (0.262–0.505)
KD	6	1450 (1238–1698)	4475 (3775–5250)	0.325 (0.322–0.328)
**HEALTHY CONTROLS**				
HCs	18	835 (795–1083)	3125 (2730–3775)	0.275 (0.250–0.303)

*IQR, interquartile range; TRAPS, tumor necrosis factor receptor-associated periodic syndrome; FMF, familial Mediterranean fever; s-JIA, systemic juvenile idiopathic arthritis; KD, Kawasaki disease; HCs, healthy controls; sTNFR, soluble tumor necrosis factor receptor*.

As shown in Figure 1B and Table 2, serum sTNFR-II levels were significantly elevated in the active phase in patients with s-JIA (median, 6,250 pg/mL; IQR 4,550–7,963) (*p* < 0.0001) and KD (median, 7,250 pg/mL; IQR 4,410–9,390) (*p* < 0.0001) compared with those in HCs (median, 3,125 pg/mL; IQR 2,730–3,775). Serum sTNFR-II levels were significantly elevated in the active phase in patients with s-JIA (*p* < 0.05) and KD (*p* < 0.05) compared with those in FMF patients (median, 3,340 pg/mL; IQR 2,375–3,858). Serum sTNFR-II levels in patients with KD were also significantly elevated in the active phase compared with those in patients with TRAPS (median, 3,100 pg/mL; 2,330–3,550) (*p* < 0.05).

As shown in Figure 1C and Table 2, the serum sTNFR-I/II ratio was significantly elevated in the active phase in patients with FMF (median, 0.401; IQR 0.349–0.497) (*p* < 0.05), s-JIA (median, 0.440; IQR 0.342–0.597) (*p* < 0.0001), and KD (median, 0.327; IQR 0.240–0.469) (*p* < 0.05) compared with that in HCs (median, 0.275; IQR 0.250–0.303), whereas this ratio was not elevated in patients with TRAPS (median, 0.297; IQR 0.282–0.382) compared with HCs.

### Comparison of Serum sTNFR-I and sTNFR-II Levels and sTNFR-I/II Ratio in TRAPS and Other Autoinflammatory Diseases in the Inactive Phase

We also measured serum sTNFR-I and sTNFR-II levels in patients with TRAPS in the inactive phase and compared these values with those obtained for patients with s-JIA, FMF, KD, and HCs. As shown in Figure 1D and Table 2, serum sTNFR-I levels were significantly lower in the inactive phase in patients with TRAPS (median, 444 pg/mL; IQR 350–495) compared with those in s-JIA patients (median, 1,040; 685–1,380) (*p* < 0.01) and KD (median, 1,450; IQR 1,238–1,698) (*p* < 0.001). Serum sTNFR-I levels in patients with TRAPS were also significantly lower compared with those in HCs (median, 835; IQR 795–1,083) (*p* < 0.05).

As shown in Figure 1E and Table 2, serum sTNFR-II levels showed no differences in the inactive phase among patients with TRAPS (median, 2,580; IQR 1,835–3,350) and FMF (median, 2,130; IQR 1,475–2,638), s-JIA (median, 2,750; IQR 2,090–3,925), KD (median, 4,475; IQR 3,775–5,250), and in HCs (median, 3,125; IQR 2,730–3,775). Serum sTNFR-II levels in patients with FMF were significantly lower compared with those in patients with KD (*p* < 0.01) and HCs (*p* < 0.05).

As shown in Figure 1F and Table 2, the serum sTNFR-I/II ratio was significantly lower in the inactive phase in patients with TRAPS (median, 0.156; IQR 0.136–0.224) compared with FMF patients (median, 0.451; IQR 0.334–0.541) (*p* < 0.01), s-JIA patients (median, 0.379; IQR 0.262–0.505) (*p* < 0.01). In contrast, serum sTNFR-I/II ratio in patients with FMF and s-JIA was significantly elevated compared with those in HCs (median, 0.275; IQR 0.250–0.303) (FMF vs. HCs, *p* < 0.01; s-JIA vs. HCs, *p* < 0.05).

### Distribution Map of Serum sTNFR-II and sTNFR-I/II Ratio

**Figure 2:**
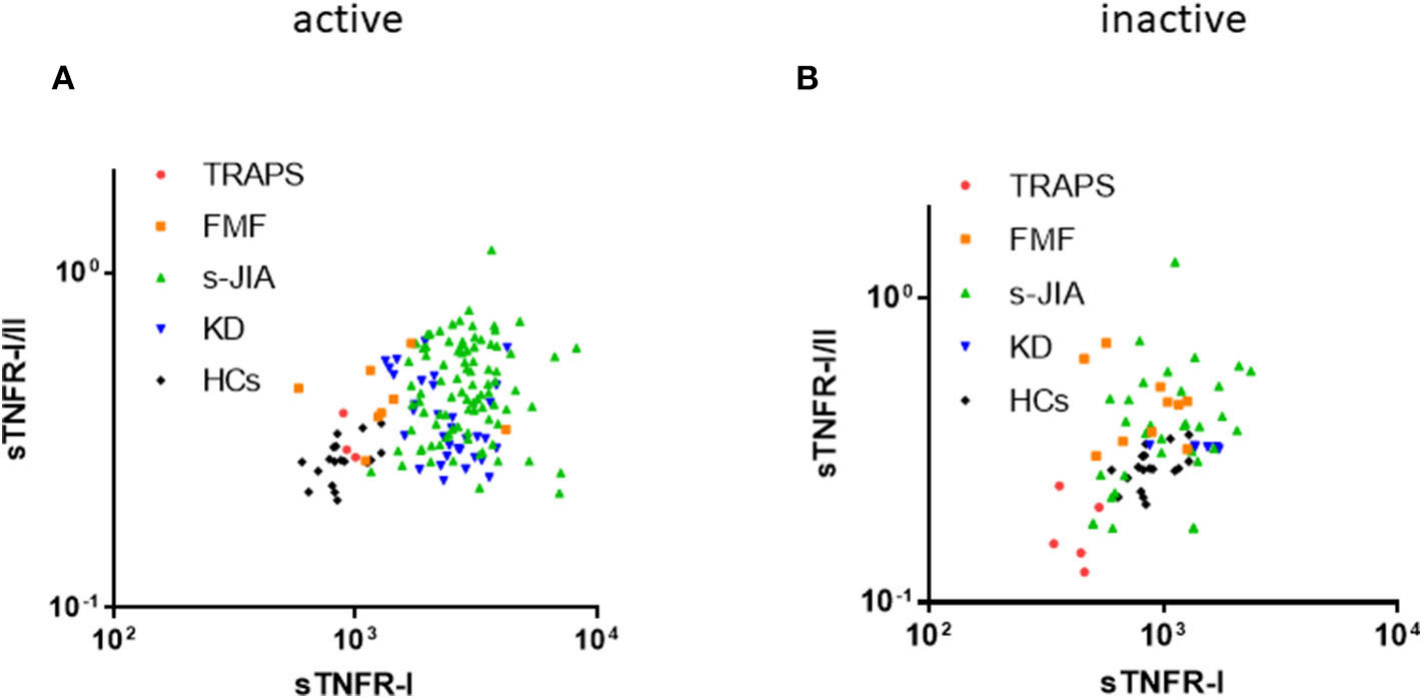
Distribution map of serum sTNFR-I and sTNFR-I/II ratio. Correlation between serum sTNFR-I levels and sTNFR-I/II ratio in each group in the active **(A)** and inactive phase **(B)**. TRAPS, tumor necrosis factor receptor-associated periodic syndrome; FMF, familial Mediterranean fever; s-JIA, systemic juvenile idiopathic arthritis; KD, Kawasaki disease; HCs, healthy controls.

As shown in Figure 2A, in the active phase, serum sTNFR-I levels and sTNFR-I/II ratio in patients with s-JIA and KD were high. In contrast, both values in TRAPS patients were similar to those in HCs. In patients with FMF, they were mildly elevated and higher than those in patients with TRAPS.

As shown in Figure 2B, in the inactive phase, serum sTNFR-I levels and sTNFR-I/II ratio in patients with TRAPS were lower than those in HCs.

## Discussion

In this study, we demonstrated that in the active phase, the serum sTNFR-I/II ratio in patients with s-JIA, KD, and FMF was significantly elevated compared with that in HCs, whereas it was not elevated in patients with TRAPS. In the inactive phase, the serum sTNFR-I/II ratio in patients with FMF and s-JIA was significantly higher compared with that in HCs, but was lower in patients with TRAPS compared with FMF and s-JIA. From these findings, low serum sTNFR-I/II ratio in the active and inactive phase might be useful for the differential diagnosis of TRAPS and other autoinflammatory diseases prior to genetic analysis for TRAPS.

TRAPS is an autosomal dominantly inherited autoinflammatory disease caused by mutations in *TNFRSF1A* (1). The pathogenesis of TRAPS remains unknown and is under investigation. One possible explanation is the shedding hypothesis (1). In normal conditions, after activation of the receptor, the extracellular region of TNFR1 is shed from the cell surface by metalloproteases. sTNFR-I effectively neutralizes TNF-α. However, increased cell surface expression of TNFR-I and decreased plasma levels of sTNFR-I were observed in patients with TRAPS, which induced increased and prolonged TNFα signaling and decreased inhibition of circulating TNFα. However, further studies demonstrated that cleavage defects were not always observed in association with TRAPS mutations. Recent studies demonstrated additional signaling defects in patients with TRAPS. Rebelo et al. showed that mutant TNFR-I may aggregate and be retained in the cytoplasm, resulting in defective cell surface expression and cell signaling (6). Furthermore, Lobito et al. revealed that mutant TNFR-I showed reduced surface expression, which was correlated with downregulated apoptosis induction and NF-κB signaling (7). The structurally altered mutant of TNFR-I failed to interact with the wild-type receptor and formed abnormal self-aggregates that were retained in the endoplasmic reticulum. Misfolding of TNFR-I in the ER induces an inflammatory response through the unfolded protein reticulum (8), ligand-independent NFκB activation (9–11), and generation of mitochondrial reactive oxygen species (12). This misfolding hypothesis might explain how the inflammatory phenotype of TRAPS may be associated with the induction of cytokines, such as IL-1β, due to an unfolded protein response.

Clinical features and laboratory parameters in patients with TRAPS often overlap with those of other autoinflammatory diseases, particularly FMF, s-JIA, and KD. Furthermore, there are no definitive biomarkers for these diseases. This situation makes the clinical diagnosis of these patients difficult. Our patients with TRAPS were initially diagnosed with s-JIA or FMF. Furthermore, the patient with TRAPS diagnosed as FMF had undetermined mutations outside of exon 10 of *MEFV*. Thus, genetic analysis is not always the best approach for diagnosing these diseases, particularly in patients with ambiguous genetic mutations. McDermott et al. reported serum s TNFR-I levels were not elevated even in the active phase in patients with TRAPS (1). However, we previously reported that serum sTNFR-I levels were significantly elevated in KD and s-JIA (13). From these findings, we hypothesized serum sTNFR-I levels might be useful for differentiating TRAPS from other autoinflammatory diseases whose clinical features are similar to TRAPS.

In this study, serum sTNFR-I levels in patients with TRAPS were not elevated even in the active phase. Furthermore, serum sTNFR-I levels were lower than those in HCs in the inactive phase. Nonetheless, serum sTNFR-II levels in patients with TRAPS did not differ from those in HCs in both the active and inactive phase. Although we examined only patients with TRAPS with T50M, C43R, and C30Y mutations of *TNFRSF1A*, McDermott et al. also reported that serum sTNFR-I levels of TRAPS patients with C33Y, T50M, C88Y, and C52F mutations of *TNFRSF1A* in the inactive phase were lower than those in HCs, and in the active phase they were more elevated than those in the inactive phase, but were not as high as levels in rheumatoid arthritis and systemic lupus erythematosus. Serum sTNFR-II levels of TRAPS patients with C33Y and C52F mutations in *TNFRSF1A* were similar between the active phase and the inactive phase (1). These findings indicate that low serum levels of sTNFR-I, both in the active and inactive phase, is a characteristic of TRAPS. Diagnosis of TRAPS is conducted via a genetic test and should be considered in suspected TRAPS patients with insufficient elevation of serum sTNFR-I levels in the active phase compared with other autoinflammatory diseases, and also with a significant decrease in these levels in the inactive phase compared with HCs.

In this study, serum sTNFR-I levels in patients with s-JIA were significantly elevated in the active phase compared with those in FMF patients. Serum sTNFR-II levels in patients with s-JIA and KD were significantly elevated in the active phase compared with those in FMF patients. The serum sTNFR-I/II ratio in patients with FMF was significantly elevated compared with that in HCs, and there were no differences in the ratio between FMF and s-JIA, and KD. These findings indicate both sTNFR-I and sTNFR-II are increased in patients with s-JIA and KD, whereas sTNFR-I is predominantly increased and sTNFR-II is not increased in patients with FMF. Furthermore, serum sTNFR-II levels in patients with FMF were significantly lower in the inactive phase compared with those in s-JIA and KD patients. During cell-mediated immune responses, sTNFR-II is mainly shed from stimulated monocytic cells and lymphocytes whereas other cells responding to IFN-γ preferentially shed sTNFR-I (14), but it is unclear why. However, monocytes/lymphocytes might contribute more to the pathogenesis of s-JIA and KD compared with that of FMF, to which neutrophils mainly contribute.

This study had some limitations. First, the sample number of TRAPS patients was very small. We measured serum levels of sTNFR-I and sTNFR-II only in TRAPS patients with T50M, C43R, and C30Y mutations in *TNFRSF1A*. Second, we did not perform a cost-benefit analysis. Third, in general, cytokine measurement by ELISA is limited to the laboratory level. Further studies to evaluate these levels in TRAPS patients with other mutations in *TNFRSF1A* are necessary. Larger studies may help to define the true diagnostic value of sTNFR-I and the sTNFR-I/II ratio as clinical markers.

In conclusion, the serum sTNFR-I/II ratio in TRAPS patients may be a useful indicator for the differentiation of TRAPS from FMF, s-JIA, and KD. Particularly, decreased serum sTNFR-I levels and sTNFR-I/II ratio in the inactive phase and not increased those in the active phase may be useful in cases of suspected TRAPS. Hence, genetic tests for TRAPS should be considered in patients with these abnormal findings.

## Data Availability Statement

The raw data supporting the conclusions of this article will be made available by the authors, without undue reservation.

## Ethics Statement

The studies involving human participants were reviewed and approved by the Institutional Review Board of Kanazawa University. Written informed consent to participate in this study was provided by the participants' legal guardian/next of kin.

## Author Contributions

JY, MS, TT, and MY were involved in the acquisition of data and analysis and interpretation of data. JY and MS wrote the manuscript. All authors were involved in the conception, design of the study, revising it critically for important intellectual content, and read and approved the final manuscript.

## Conflict of Interest

The authors declare that the research was conducted in the absence of any commercial or financial relationships that could be construed as a potential conflict of interest.
